# Substituting Sodium Hydrosulfite with Sodium Metabisulfite Improves Long-Term Stability of a Distributable Paper-Based Test Kit for Point-of-Care Screening for Sickle Cell Anemia

**DOI:** 10.3390/bios7030039

**Published:** 2017-09-20

**Authors:** Kian Torabian, Dalia Lezzar, Nathaniel Z. Piety, Alex George, Sergey S. Shevkoplyas

**Affiliations:** 1 Department of Biomedical Engineering, University of Houston, 3605 Cullen Blvd, Houston, TX 77204-5060, USA; ktorabian@gmail.com (K.T.); dalia.lezzar94@gmail.com (D.L.); nate.piety@gmail.com (N.Z.P.); 2 Department of Pediatrics, Section of Hematology-Oncology, Baylor College of Medicine, Houston, TX 77030, USA; axgeorge@txch.org

**Keywords:** sickle cell anemia, paper-based diagnostics, point-of-care screening

## Abstract

Sickle cell anemia (SCA) is a genetic blood disorder that is particularly lethal in early childhood. Universal newborn screening programs and subsequent early treatment are known to drastically reduce under-five SCA mortality. However, in resource-limited settings, cost and infrastructure constraints limit the effectiveness of laboratory-based SCA screening programs. To address this limitation our laboratory previously developed a low-cost, equipment-free, point-of-care, paper-based SCA test. Here, we improved the stability and performance of the test by replacing sodium hydrosulfite (HS), a key reducing agent in the hemoglobin solubility buffer which is not stable in aqueous solutions, with sodium metabisulfite (MS). The MS formulation of the test was compared to the HS formulation in a laboratory setting by inexperienced users (*n* = 3), to determine visual limit of detection (LOD), readout time, diagnostic accuracy, intra- and inter-observer agreement, and shelf life. The MS test was found to have a 10% sickle hemoglobin LOD, 21-min readout time, 97.3% sensitivity and 99.5% specificity for SCA, almost perfect intra- and inter-observer agreement, at least 24 weeks of shelf stability at room temperature, and could be packaged into a self-contained, distributable test kits comprised of off-the-shelf disposable components and food-grade reagents with a total cost of only $0.21 (USD).

## 1. Introduction

Sickle cell disease (SCD) refers to a group of common recessively inherited hemoglobin disorders associated with significant lifelong morbidities and premature mortality. SCD results from the inheritance of a mutated allele coding for the β-globin subunit of hemoglobin along with another mutated allele coding for the same or another aberrant form of hemoglobin [1,2]. This mutated β-globin allele results in the production of sickle hemoglobin (HbS), a form of hemoglobin (Hb) that polymerizes when deoxygenated, unlike normal adult hemoglobin (HbA). The polymerization of deoxy-HbS molecules into rigid fibers deforms the red blood cell (RBC) membrane and causes the characteristic ‘sickled’ shape of affected sickle RBCs. Sickle RBCs are markedly less deformable and more fragile than normal, healthy RBCs, which causes them to occlude blood vessels, cause chronic ischemia-reperfusion injury, and lyse frequently, resulting in severe anemia, chronic painful episodes, and predisposition to infection [3,4].

When the allele coding for HbS is inherited heterozygously with the allele coding for HbA (i.e., genotype *HbAS*) it causes what is known as sickle cell trait (SCT) where both forms of Hb are produced and individuals are typically healthy, do not need medical intervention related to HbS, and may have improved resistance to parasites such as *Plasmodium falciparum* [3,4]. In Angola, for example, the HbS allele is present in over 20% of the population, with about 98% of those carriers having the allele heterozygously [5,6]. However, when the HbS allele is inherited homozygously (i.e., genotype *HbSS*), it causes sickle cell anemia (SCA), a condition in which 40–100% of all Hb produced is HbS and individuals experience some of the most severe symptoms associated with SCD. Homozygous inheritance of the HbS allele and an allele coding for another non-HbS, aberrant form of Hb results in other, less common forms of SCD which have varying degrees of clinical severity [7,8]. For the purposes of this study, we will focus on the differentiation of SCA—the most common form of SCD—from SCT and normal, healthy individuals (i.e., genotype *HbAA*).

The mortality rate for SCA is the highest in the first five years of a child’s life, as young children are prone to acute infections and splenic sequestration crises [9]. Improvements in survival rates for SCA over the last few decades have been largely due to earlier diagnosis and the initiation of simple, inexpensive prophylaxis, such as penicillin, to combat opportunistic infections. Almost all children born in the United States, and many other resource-rich countries, are screened for SCA at birth as part of universal newborn screening programs that test for diseases and conditions where early initiation of treatment is crucial for survival and healthy, normal development [10]. However, due to cost and infrastructure limitations, this type of universal screening is oftentimes not feasible in the resource-limited settings where the incidence of HbS is highest, such as in sub-Saharan Africa, where approximately 80% of global individuals with SCA are born [2,4,11]. Nearly all children with SCA who are diagnosed soon after birth and promptly given treatment survive into adulthood, while the mortality rates for undiagnosed (and, therefore, untreated) children in sub-Saharan Africa are estimated to be as high as 50–90% by the age of five years [2,11].

The implementation of wide-spread screening and follow-up care for individuals with SCA has been predicted to potentially save the lives of up to 10 million children by 2050 in the countries most affected by the disease (e.g., Nigeria, Angola, Democratic Republic of the Congo, India) [7]. Though highly successful, most of the pilot screening programs in these settings have used conventional diagnostic methods such as high-performance liquid chromatography (HPLC) or isoelectric focusing electrophoresis (IEF) to identify SCA, both of which are relatively high-cost, technically complex, and dependent on stable infrastructure [12,13]. The requirements and limitations of these conventional diagnostic methods limit screening to those born in major health centers, and prevent life-saving screening efforts from reaching more remote, less well-equipped community health outposts and those born out-of-hospital [13,14,15,16].

In order to address these limitations, our research group recently developed a simple, equipment- and electricity-free, paper-based test capable of differentiating normal (*HbAA*), SCT (*HbAS*), and SCA (*HbSS*) blood samples [17,18]. The paper-based test was previously shown to be accurate (93% sensitivity, 94% specificity for differentiating SCA), low-cost ($0.07 in material and reagent costs), robust and easy to use (Angolan health workers were proficient in performing the test after one demonstration), and rapid (less than 30 min from start to readout) [19]. However, the previously-developed version of the paper-based SCA screening test required the use of a reducing agent—sodium hydrosulfite (HS)—which quickly becomes oxidized in aqueous solutions (especially when exposed to oxygen) [18,20]. The relative instability and volatility of HS made shipping and long-term storage of the previous version of the test impractical [19,20]. Here we describe how replacing HS with a higher concentration of sodium metabisulfite (MS) addressed the limitations of the previously-developed test without negatively impacting test performance. In this study we found that, compared to the previous test, the novel MS formulation of the paper-based SCA screening test has a superior limit of detection (10% vs. 20% HbS of total Hb content), faster readout time (21 min vs. 30 min), better diagnostic performance (97.3% sensitivity, 99.5% specificity for differentiating SCA), high intra- and inter-observer agreement (κ = 0.93, κ = 0.87 respectively), longer shelf stability at room temperature (at least 24 weeks vs. one week for aqueous storage conditions), and could be packaged into a self-contained, distributable test kit with a total cost of only $0.21 (USD).

## 2. Materials and Methods

### 2.1. Blood Samples

The study protocol was approved by the University of Houston and Baylor College of Medicine institutional review boards. Venous whole blood samples were obtained with written informed consent in 4 mL Vacutainer tubes (K_2_EDTA, BD, Franklin Lakes, NJ, USA) from healthy, normal volunteers and patients of the Texas Children’s Hematology Centers (Houston, TX, USA). Blood samples were stored at 4 °C and used within two months following collection. The sickle hemoglobin (HbS) content of blood samples from individuals with sickle cell anemia (SCA) was quantified with high-performance liquid chromatography (HPLC; Primus Ultra Variant, Trinity Biotech, Wicklow, Ireland). Healthy volunteers were assumed to have 0% HbS. Hematocrit (Hct) and hemoglobin concentration ([Hb]) were measured with an automated hematology analyzer (Medonic, Boule Medical AB, Spånga, Sweden), and ABO-RhD blood type was determined using blood type test kits (EldonCard, Eldon Biologicals, Gentofte, Denmark).

Artificially-reconstituted blood samples with specific Hct (22%—a physiological Hct for SCA) and HbS concentrations (0, 10, 20, 40, and 80% HbS) were made by combining type-matched blood samples from normal (*HbAA*) and SCA (*HbSS*) individuals. Sample Hct was adjusted via centrifugation at 500× *g* for 10 min (Beckman Microfuge 22R, Beckman Coulter, Brea, CA, USA) and reconstitution of red blood cell (RBC) sediment in autologous plasma. The type-matched and Hct-matched *HbAA* and *HbSS* blood samples were then combined at various ratios according to the following equation:

(1) $$\%HbS=\frac{({\left[Hb\right]}_{HbSS})\left(V_{HbSS}\right)\left(\%HbS_{HbSS}\right)}{({\left[Hb\right]}_{HbSS})\left(V_{HbSS}\right)\;+\;({\left[Hb\right]}_{HbAA})\left(V_{HbAA}\right)},$$

where V is the volume, subscripted *HbSS* refers to samples from patients with SCA, and subscripted *HbAA* refers to samples from healthy, normal volunteers.

For this study, venous blood samples were collected as described above. For the self-contained SCA screening test kit, capillary blood can be collected via finger or heel prick. Capillary and venous blood have both been shown to produce comparable diagnostic results for the assay [18,19,20]. Additionally, the HS version of the paper-based SCA screening test has been previously shown to be robust against variations in Hct and associated variations in [*Hb*] within the physiological range [18,19]. Physiological concentrations of fetal hemoglobin (*HbF*) amongst infants, adults, and hydroxyurea patients were also shown to not impact test performance [18,19].

Blood samples used to perform the study of test kit stability were replaced with new samples—with the same Hcts and HbS concentrations—after 20 days of storage at 4 °C in order to counteract any potential effects due to storage-based RBC and/or Hb deterioration (i.e., storage lesion).

### 2.2. Hemoglobin Solubility Buffers

The hemoglobin solubility buffers compared in this study each consisted of three components: saponin (Sigma-Aldrich, St. Louis, MO, USA), a reducing agent, and a concentrated phosphate buffer [17,18,19,20]. Potassium phosphate buffer at 2.49 M was made by dissolving solid 1.24 M (169 g/L) monobasic and 1.25 M (217 g/L) dibasic potassium phosphate (Sigma-Aldrich, St. Louis, MO, USA) in deionized water. Saponin (4 g/L) was added to irreversibly lyse RBCs by creating holes in the lipid bilayer through sequestration of cholesterol, thereby releasing hemoglobin into the buffer [19]. The saponin used in this study was obtained from Quillaja bark (sapogenin content not less than 10%) and contained impurities which made the powder hygroscopic [21]. The reducing agent—either HS (30 g/L; 3% *w*/*v*; Sigma-Aldrich, St. Louis, MO, USA) or MS (100, 150 or 200 g/L; 10, 15, 20% *w*/*v*; Sigma-Aldrich, St. Louis, MO, USA)—then converts the released Hb into deoxy-Hb, which is either soluble (e.g., deoxy-HbA, deoxy-HbF, deoxy-HbC) or insoluble (deoxy-HbS) in the phosphate buffer [17,18,19,20]. Additionally, buffer consisting of food-grade MS (Duda Energy LLC, Decatur, AL, USA) and food-grade saponin (Desert King, San Diego, CA, USA) was made using the same concentrations as above.

The stability of the MS and HS solubility buffers was compared under two storage conditions: ‘wet’ and ‘dry’. Dry refers to the saponin and MS or HS being stored in dry powdered form and mixed with concentrated phosphate buffer on the day of the experiment, while wet refers to dry reagents premixed with concentrated phosphate buffer and stored until the day of the experiment. Individual sets of reagents were stored within heat-sealed polyethylene-lined 2 mm thick foil pouches (Xin Jiu Technology, Taoyuan, Taiwan) to prevent exposure of reducing agents and hygroscopic compounds in saponin to oxygen and humidity outside of the packaging. The reagents were stored and tested at room temperature (18–26 °C, 30–50% relative humidity). The buffers were stored between one and 166 days before use. Results from tests performed using stored buffers were compared against those for reagents prepared on the same day of the experiment from dry powdered ingredients stored in their original containers.

### 2.3. Design and Operation of the Paper-Based Test

**Figure 1:**
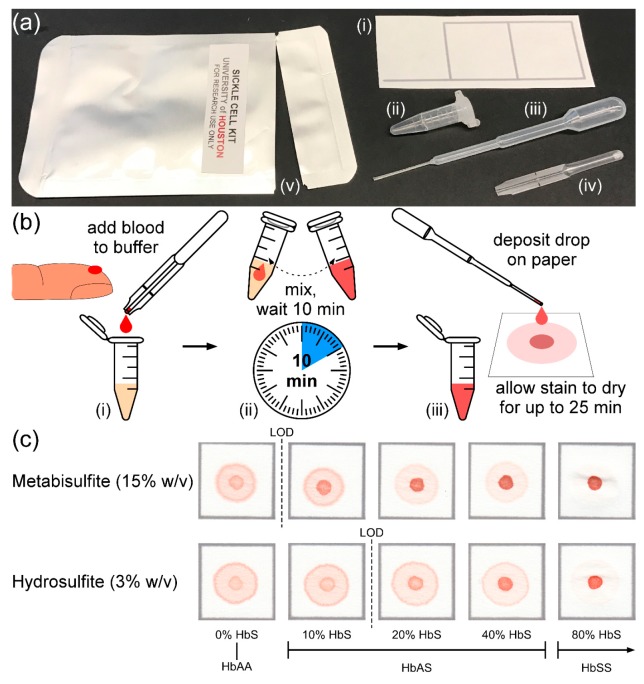
Overview of the distributable paper-based SCA diagnostic test kit. (**a**) Photograph of the sickle cell kit with all components necessary to perform the test: (i) patterned chromatography paper; (ii) reagent tube containing reagents; (iii) reagent dropper; (iv) blood dropper; and (v) foil pouch. (**b**) Schematic illustration showing the steps required to perform the paper-based test: (i) ~20 μL of whole blood is collected via finger-stick using the blood dropper and deposited in the reagent tube; (ii) the blood is mixed with a preset volume of buffer (containing either sodium hydrosulfite or sodium metabisulfite) via manual agitation; and (iii) after 10 min, a drop (~20 μL) of the mixture is deposited on the chromatography paper using the reagent dropper and allowed to dry for up to 25 min before being evaluated visually. (**c**) Representative bloodstains produced by the metabisulfite (top) and hydrosulfite (bottom) versions of the paper-based test for samples with various sickle hemoglobin (HbS) concentrations. The limit of detection (LOD) for each version is indicated by a dashed line. Typical HbS concentration ranges for adults and children older than six months of age with different genotypes (normal—*HbAA*; SCT—*HbAS*; SCA—*HbSS*) are marked below the stains.

The design and operation of the paper-based SCA screening test have previously been described in detail [17,18,19,20]. Briefly, 20 µL of whole blood is collected and mixed with Hb solubility buffer by inversion, the blood and buffer mixture is allowed to incubate at room temperature for 10 min, 20 µL of blood is then dropped onto chromatography paper (Whatman Chr 1, Sigma-Aldrich, St. Louis, MO, USA) and allowed to dry (Figure 1b). Insoluble deoxy-HbS polymers, if present, become entangled in the paper substrate and form a dark red spot in the center of the stain, while soluble forms of Hb wick laterally through the paper pores and produce a more diffuse pink ring (Figure 1c).

### 2.4. Blood Stain Pattern Interpretation

Blood stain patterns were interpreted visually by eye and/or digitized using a portable scanner (CanoScan LiDE110, Canon USA, Lake Success, NY, USA) and quantified using a custom image analysis algorithm implemented in MATLAB (The MathWorks, Natick, MA, USA). Inexperienced users (*n* = 3) with little to no experience performing and interpreting the paper-based test were provided with a set of representative images of blood stain patterns resulting from both the MS and HS versions of the test performed using normal, SCT, and SCA blood samples (using Hb solubility buffer prepared on the same day of the experiment from dry powdered ingredients stored in their original containers). The previously described ‘S-index’—defined as the quotient of the mean red color intensity of pixels in the center spot area of the blood stain and the mean red color intensity of pixels in the ring area of the blood stain (red color intensity = 255 − B, where B is the blue channel of the RGB values for the digitized image)—was used to quantify the differences between blood stain patterns produced by different formulations of the test [18].

### 2.5. Test Performance and Statistical Analysis

Test performance metrics were calculated as: Sensitivity = TP/(TP + FN); specificity = TN/(FP + TN); positive predictive value (PPV) = TP/(TP + FP); negative predictive value (NPV) = TN/(TN + FN); and accuracy = (TP + TN)/(TP + FP + TN + FN), where TP = true positive, FP = false positive, TN = true negative, and FN = false negative. Fleiss’ kappa statistic was used to assess intra- and inter-operator agreement for visual scoring of blood stains [22,23]. Mean, standard deviation, *p*-values, and confusion matrices were calculated using built-in functions in MATLAB 2014b (The MathWorks, Natick, MA, USA).

## 3. Results

We hypothesized that we could improve the stability and performance of our previously-developed paper-based screening test for SCA by replacing a key component of the Hb solubility buffer, sodium hydrosulfite (HS), with sodium metabisulfite (MS), a chemically stable food additive. The concentration of MS used to replace HS was determined by comparing the previously described S-index—defined as the quotient of the red color intensities of the center area (proportional to the amount of HbS) and the ring area (proportional to other forms of Hb) of the blood stain—for buffer formulations with 10, 15, and 20% MS (*w*/*v*) to the S-index for the previously-developed buffer formulation with 3% HS (*w*/*v*) for a set of samples with HbS concentrations from 0 to 40% HbS [18]. 15% MS (*w*/*v*) was chosen as the final formulation for the Hb solubility buffer because it produced the greatest difference in the S-Index between 0 and 20% HbS, and the most gradual change in S-Index over the range of 20–40% HbS. The Hb solubility buffer containing 15% MS (*w*/*v*) was used to perform all experiments described in this study.

### 3.1. Limit of Detection

The limit of detection (LOD) for this test is defined as the lowest percentage of HbS (out of the total amount of Hb in a sample) that will produce a blood stain on paper which is visually distinguishable from characteristic blood stains for samples without HbS (0%). The previously developed HS test is capable of identifying SCA in adults and children older than six months of age and has a reported LOD of ~15% HbS [19]. To determine the LOD of the MS version of the test, inexperienced users (*n* = 3) were asked to visually score a set of images of blood stains in paper (*n* = 370) produced by the MS formulation of the test (370 stains × 3 users = 1110 total scores) as either HbS-negative (HbS = 0%) or HbS-positive (HbS > 0%). The inexperienced users correctly scored 820 of the 888 blood stain images with ≥10% HbS (92.3%) as having some HbS and 220 of the 222 blood stain images with <10% HbS (99.1%) as having no HbS. These results suggest that, when evaluated visually by an inexperienced user, the LOD of the MS formulation of the paper-based SCA screening test was ~10% HbS. This LOD was confirmed to produce the greatest AUC (area under the curve) on an ROC (receiver operating characteristic) curve.

### 3.2. Test Readout Time

After the mixture of blood and Hb solubility buffer is deposited on chromatography paper, it takes approximately 25 min for the blood stain to become completely dry. However, accurate visual diagnoses can be made from blood stain patterns before they are completely dry. Inexperienced users (*n* = 3) were asked to visually score blood stains for unknown samples every minute for 25 min as the stains dried following deposition of the mixture onto paper. Samples with 0% HbS (normal) were correctly scored by all three users after 7 min of drying time, samples with HbS levels characteristic of SCT (10–40% HbS) were correctly scored by all users after 11 min, and samples with HbS levels characteristic of SCA (>40% HbS) could be scored correctly after 1 min. These results suggest that the paper-based SCA screening test can be performed and interpreted within 21 min (10 min preparation and incubation +11 min drying before readout).

### 3.3. Test Performance Metrics

**Figure 2:**
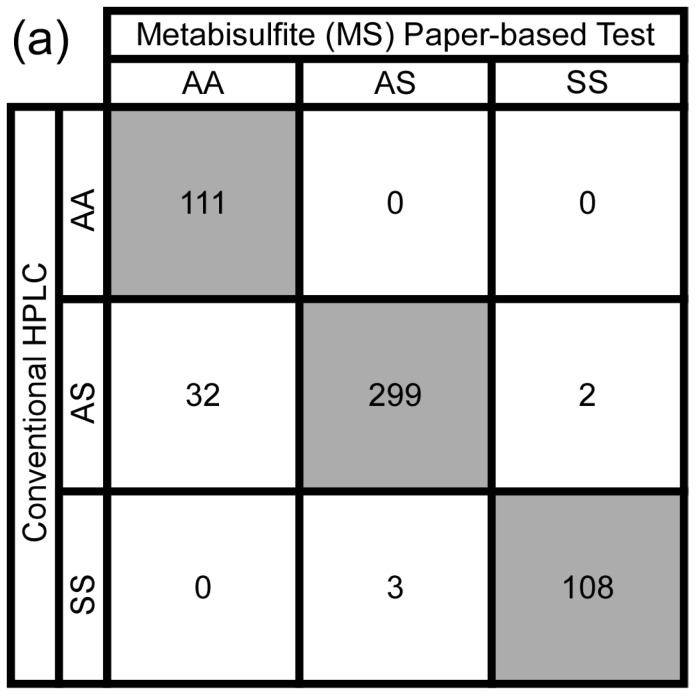

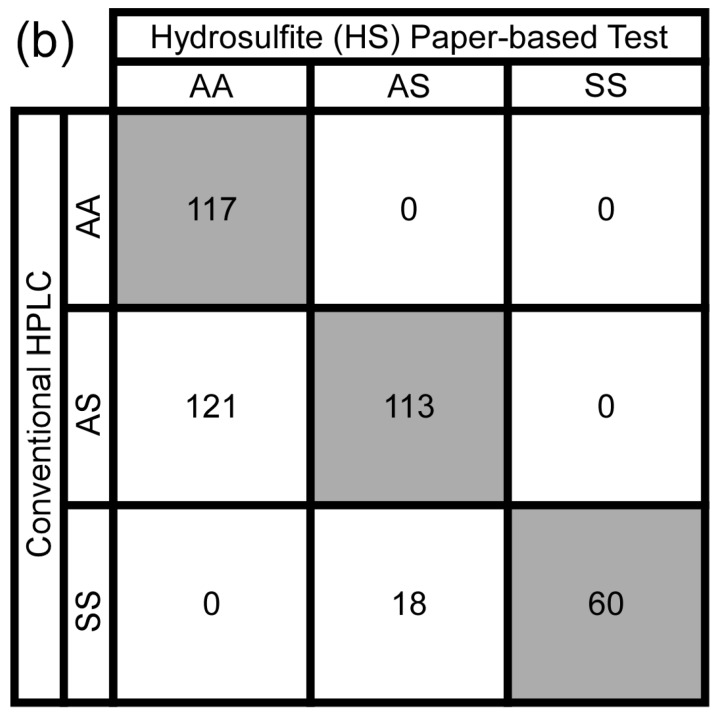
Diagnostic accuracy of the paper-based SCA screening test kit. (**a**) Aggregate confusion matrix for screening of blood samples (*n* = 185) with characteristic HbS concentrations via visual interpretation (inexperienced users; *n* = 3) of the blood stains produced on paper by the metabisulfite (MS) formulation of the test (185 samples × 3 users = 555 total scores). (**b**) Aggregate confusion matrix for the screening of blood samples (*n* = 143) with characteristic HbS concentrations via visual interpretation (inexperienced users; *n* = 3) of the blood stains produced on paper by the hydrosulfite (HS) formulation of the test (143 samples × 3 users = 429 total scores). Rows correspond to characteristic genotypes (based on HbS concentration measured using conventional high-performance liquid chromatography—HPLC) and columns correspond to predicted genotypes (diagnosed by the paper-based test). Shaded cells contain the numbers of correct diagnoses.

To determine the performance of the MS version of the test, inexperienced users (*n* = 3) were asked to visually score a set of images of blood stains on paper (*n* = 185) produced by the MS version of the test as either normal (*HbAA*; characteristic HbS = 0%), SCT (*HbAS*; characteristic HbS = 10–40%) or SCA (*HbSS*; characteristic HbS > 40%) by comparing them to a set of representative blood stain images from each category. Figure 2 shows the aggregate confusion matrices of the visual diagnoses made by the three inexperienced users with both the MS and HS versions of the paper-based test. Using the MS formulation inexperienced users could visually distinguish between blood samples with no HbS and blood samples with ≥10% HbS (i.e., *HbAA* vs. *HbAS* and *HbSS*) with 92.8% sensitivity, 100% specificity, 100% positive predictive value (PPV), 77.6% negative predictive value (NPV) and 94.2% overall diagnostic accuracy (Figure 2a). Users could also distinguish between blood samples with ≥80% HbS (characteristic of SCA) from blood samples with <80% HbS (characteristic of normal and SCT) (i.e., *HbSS* vs. *HbAA* and *HbAS*) with 97.3% sensitivity, 99.5% specificity, 98.2% PPV, 99.3% NPV, and 99.1% overall diagnostic accuracy (Figure 2a).

When the same three inexperienced users were asked to score blood stains on paper (*n* = 143) produced by the previously-developed HS formulation of the test, they could visually distinguish between blood samples with no HbS and blood samples with ≥10% HbS (i.e., *HbAA* vs. *HbAS* and *HbSS*) with 61.2% sensitivity, 100% specificity, 100% PPV, 49.2% NPV, and 71.8% overall diagnostic accuracy (Figure 2b). Users could also distinguish between blood samples with ≥80% HbS (characteristic of SCA) from blood samples with <80% HbS (characteristic of normal and SCT) (i.e., *HbSS* vs. *HbAA* and *HbAS*) with 76.9% sensitivity, 100% specificity, 100% PPV, 95.1% NPV, and 95.8% overall diagnostic accuracy (Figure 2b).

### 3.4. Intra- and Inter-Observer Agreement

The Fleiss’ kappa statistical measure (κ) for assessing intra-observer agreement of the scoring of blood stains on paper (*n* = 370) produced by the MS version of the test was κ = 0.93 ± 0.04 (individual scores: 0.88, 0.93, and 0.97), which suggests that there was almost perfect self-consistency for each of the three inexperienced test users. The Fleiss’ kappa statistical measure for assessing inter-observer agreement for the MS version of the test was κ = 0.87, which suggests that there was almost perfect agreement between the three inexperienced test users. When the same three inexperienced users were asked to score blood stains on paper (*n* = 286) produced by the previously-developed HS formulation of the test, κ = 0.85 ± 0.09 (individual scores: 0.72, 0.88, and 0.94) for intra-observer agreement and κ = 0.75 for inter-observer agreement. These results suggest that there is a very high level of intra- and inter-observer agreement for visual diagnosis of SCT and SCA using the HS version of the test, but that both intra- and inter-observer agreement are better for the MS version of the test.

### 3.5. Test Kit Stability

**Figure 3:**
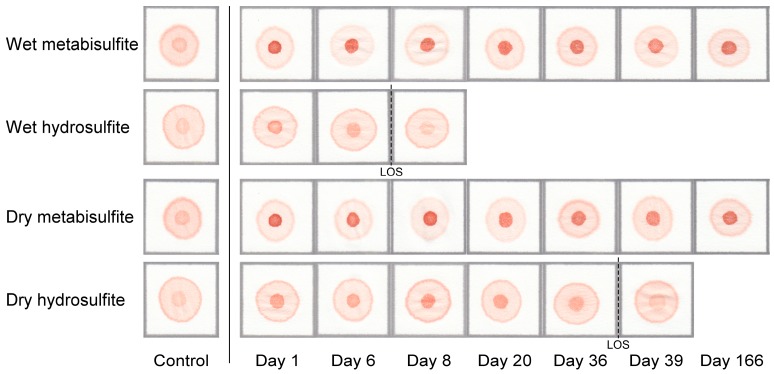
Representative images of blood stains demonstrating the reagent stability of the metabisulfite (MS) and hydrosulfite (HS) formulations of the paper-based SCA screening test under wet and dry storage conditions. (**left**) Blood stain images for samples with 0% HbS (control) produced by the MS and HS versions of the test using freshly prepared reagents. (**right**) Blood stain images for samples with 20% HbS (limit of detection of HS version of test) produced by the MS and HS versions of the test using reagents stored under wet (MS powder mixed with aqueous buffer components before storage) or dry (MS powder stored separately from aqueous buffer components) conditions. The limit of stability (LOS)—i.e., the maximum amount of time reagents can be stored before the difference in pattern between samples with 0% HbS and samples with HbS concentrations greater than, or equal to, the limit of detection of tests performed using fresh reagents becomes statistically insignificant—is indicated by a dashed line.

Figure 3 shows the stability of the reagents comprising the Hb solubility buffer for the MS and HS formulations of the paper-based test when stored under dry (MS powder stored separately from aqueous buffer components) or wet (MS powder mixed with aqueous buffer components before storage) conditions over the course of 24 weeks. Reagents were considered stable as long as the difference in the S-index—defined as quotient of the red color intensities of the center area and ring area of the blood stain—between samples with 0% HbS and samples with HbS concentrations greater than, or equal, to the LOD of tests performed using fresh reagents (i.e., ≥10% HbS for MS and ≥20% HbS for HS) remained statistically significant (*p* < 0.05).

The MS formulation maintained stability over the course of all 24 weeks studied regardless of whether MS was stored under dry or wet conditions. The difference between blood stain patterns for samples with 0% HbS and ≥10% HbS was still visually obvious and statistically significant (*p* < 0.05) at the end of the 24-week study period (Figure 3). When stored under dry conditions the HS formulation remained 100% stable until day 36 (~5 weeks) of the study period, after which it lost all activity—i.e., the difference in blood stain patterns for samples with 0% HbS and ≥20% HbS was not visually obvious or statistically significant (*p* > 0.05). When stored under wet conditions the HS formulation remained 100% stable until day 6 (~1 week) of the study period, after which it lost all activity (Figure 3). These results suggest that, regardless of whether MS is stored in dry or wet form, the MS version of the test has a shelf life which is (at the least) ~5 times as long as the shelf life for a dry HS version of the test and ~24 times as long as the shelf life for a wet HS version of the test. Additional testing showed that, when stored under wet conditions, the MS formulation remained 100% stable for (at least) one week when stored at 62 °C (an upper bound on hot temperatures reached in storage in desert climates [24]).

### 3.6. Distributable Test Kit Cost

A self-contained, distributable kit containing all reagents and materials necessary to perform the paper-based SCA screening test as described above was assembled using the following commercially-available, off-the-shelf components: blood dropper (Microsafe 20 μL, Safe-Tec, Ivyland, PA, USA), reagent tube (0.5 mL microcentrifuge tube, Sigma-Aldrich, St. Louis, MO, USA), reagent dropper (Disposable Graduated Transfer Pipet, VWR, Radnor, PA, USA), chromatography paper (Whatman Chr 1, Sigma-Aldrich, St. Louis, MO, USA), and a foil polyethylene-lined pouch (Xin Jiu Technology, Taoyuan, Taiwan) (Figure 1a). The key reagents used in this test kit were food-grade: sodium metabisulfite (Duda Energy, Decatur, AL, USA) and saponin (Desert King, San Diego, CA, USA). Table 1 shows a cost breakdown for the reagents and materials comprising the self-contained, distributable paper-based SCA screening test kit. The total cost of all materials, reagents and packaging necessary to perform the paper-based SCA screening test using this kit was $0.21 (USD).

## 4. Discussion

Universal screening for SCA using conventional, laboratory-based methods (e.g., IEF, HPLC) is currently unfeasible in many resource-limited settings because of the prohibitively high cost and lack of access to the technical infrastructure required to support such testing. The advent of a simple, stable, equipment-free, visually-interpreted and inexpensive screening test for SCA could greatly improve the survival rate of the hundreds of thousands born with the disease each year, by enabling an earlier initiation of simple and effective prophylactic care. The point-of-care screening technology described in this study has the potential to significantly decrease the cost and technical complexity of implementing universal screening programs in resource-limited regions where SCA has the highest incidence [7].

Recently, multiple research groups have developed screening tests for sickle cell disease intended for use in resource-limited settings [25,26,27,28]. Some of the most promising emerging technologies include an antibody-based lateral flow assay that can detect HbS, HbA, and HbC and an aqueous multiphase system used to separate dense sickle RBCs from healthy RBC populations with lower density [25,26,27,29]. However, none of the current or developing tests adequately address all of the unique design requirements necessary to create a test which can be distributed, stored, performed, and interpreted in resource-limited settings. For example, antibody-based tests are notoriously prone to stability and reproducibility issues when taken outside the laboratory, since antibodies generally denature above 37 °C (mammalian body temperature) and are rarely specific (nonspecific antibodies may bind unintended antigens to produce a false positive signal) [26]. However, the antibody-based SCD assay is simple to use, has shown >98% sensitivity and specificity in a laboratory setting for identifying *HbSS* blood, can accurately determine the presence of HbC (100% sensitivity and specificity) and has the potential to be very effective at identifying other non-HbS hemoglobin variants [25,26]. As a result, the antibody-based SCD assay is most appropriate for situations where high genotypic resolution (i.e., the ability to specifically identify different SCD genotypes) is needed, but may be constrained from a cost and long-term stability perspective. The density-based test, on the other hand, is highly sensitive to changes in reagent properties (e.g., change in density due to evaporation) and is not equipment- and electricity-free (i.e., requires a car-battery powered centrifuge) [29]. The complexity of the density based test, and its sensitivity to comorbidities and other factors that influence RBC density, such as high amounts of HbF in newborns, resulted in lower sensitivity (86%) and specificity (60%) for identifying *HbSS* blood in resource-limited settings in Zambia [29]. Furthermore, the density-based test has not been able to distinguish *HbAS* from *HbAA* [27,29]. As such, none of the technologies currently on the market or in public development fully address the unmet need for a truly simple, stable, and low-cost SCA screening test.

Our research laboratory has previously attempted to meet this need by developing and validating a paper-based SCA screening test capable of sensitive and specific differentiation of SCA from SCT and normal individuals in adults and children older than six months of age [17,19]. However, the Hb solubility buffer on which the previously-developed test is based utilized a reducing agent—sodium hydrosulfite (HS)—which is expensive to ship and difficult to store due to its combustibility as a solid (Hazmat Class 4.2—spontaneously combustible) and fast oxidation in aqueous solutions (t_1/2_ < 1 day at 25 °C) [30]. Here we presented the development and characterization of an alternative formulation of the Hb solubility buffer that eliminates the need for HS via substitution of an optimized concentration of sodium metabisulfite (MS)—a common preservative used in the food, textile, and photography industries. MS is approximately half the price of HS when purchased commercially, is not a regulated substance (i.e., does not need to be shipped as hazardous goods), and is stable for up to a year as a solid at room temperature and at least ~6 months in solution with exposure to oxygen [30].

The MS formulation of the paper-based SCA screening test described here successfully addresses many of the technical and logistical problems of the conventional approaches described above as well as the limitations of the previously developed HS formulation. Firstly, the use of MS instead of HS increased the shelf-life of the aqueous Hb solubility buffer by up to ~24× (Figure 3), thus allowing for the packaging of a distributable kit containing premade buffer with a reasonably long shelf-life. The MS formulation maintained stability over the course of a 24-week period regardless of storage under dry or wet conditions—i.e., the difference in the blood stain pattern between samples with 0% HbS and ≥10% (the LOD for MS tests performed using fresh reagents) HbS was still visually obvious and statistically significant (*p* < 0.05) at the end of the study period (Figure 3). In contrast, the HS formulation only remained stable for ~5 weeks under dry conditions and ~1 week under wet conditions (Figure 3). Additional experiments showed that the MS formulation of the test remained stable for at least one week when stored under wet conditions at 62 °C (an upper bound on hot temperatures reached in desert climates on Earth [24]). The improved storage capability of the MS-based assay will be crucial for the expansion of SCA screening programs in regions without established supply chains and clinical facilities.

Secondly, in addition to improving the stability of the paper-based SCA test, the substitution of MS for HS improved the LOD of the assay by two-fold, allowing for the detection of as little as 10% HbS in a blood sample, compared to 15% HbS with HS. Inexperienced users visually differentiated SCA from normal or SCT samples with 97.3% sensitivity, 99.5% specificity, and almost perfect agreement between scorers (Figure 2a). In comparison, when using the HS test, users were only able to visually differentiate SCA with 76.9% sensitivity and 100% specificity (Figure 2b). Given the higher concentration of metabisulfite used to achieve blood stain color intensities similar to the HS test, this improvement in LOD could be partially due to the increase in Hb solubility buffer osmolality, which causes more HbS to precipitate out of the solution and form a darker stain center spot on the paper [18]. It has previously been shown that HS deoxygenates HbS rapidly, resulting in the formation of amorphous precipitates of short, randomly-oriented Hb fibers, while MS has been shown to deoxygenate Hb more gradually, resulting in the formation of long, organized Hb polymers characteristic of SCA [31,32]. These comparatively larger deoxy-HbS aggregates formed using MS are more likely to become entangled within the chromatography paper pores—causing a higher fraction of total deoxy-HbS to be retained in the area of the initial drop (center spot)—compared to the smaller, more irregular aggregates formed using HS, which are more likely to be wicked laterally through the paper pores toward the edge of the stain (peripheral ring). Therefore, an alternate explanation for the improvement in LOD could be that the larger fraction of total deoxy-HbS retained in the area of the initial drop, as a result of using MS compared to HS, produces center spots which are both darker (higher absolute amount of deoxy-HbS in the center spot) and more well-defined (lower fraction of deoxy-HbS in peripheral ring) [33]. This improvement in signal detection is an important development for screening young children, who produce less HbS (due to increased HbF (fetal Hb) production) than is observed in typical adult Hb profiles.

Additionally, the MS formulation of the test can be performed and interpreted more rapidly than the HS formulation. Inexperienced users made accurate visual diagnoses from blood stain patterns within 11 min of deposition onto chromatography paper (i.e., before the blood stains on the paper were fully dry) which corresponds to 21 min from the start of the test. This is an improvement over the reported readout time of 30 min for the HS test. The more rapid availability of test results could permit immediate clinical intervention at the point-of-care and could potentially enable counseling of families with SCA newborns before discharge. The ability to deliver results during the first visit to a health clinic is vital because in regions which lack established communication infrastructure, re-contact and follow-up rates for affected newborns have been reported to be as low as 50% [5].

Finally, using MS allowed for the design of a self-contained, low-cost test kit comprised of easily-accessible, food-grade reagents (not an option for HS) and off-the-shelf disposable plastic components with a total cost of $0.21 (USD) (Table 1). The test kit consists of a blood dropper, reagent dropper, reagent tube, food grade reagents, chromatography paper and a foil pouch (Figure 1a), with >66% of the total cost being accounted for by the disposable plastic droppers. Switching from HS to MS also decreased the amortized cost of the test significantly (primarily due to shipping restrictions on HS). The transportation, storage, and material cost savings of this MS-based test, as well as the wide availability of food-grade MS, make the SCA screening test a tangible reality in even the most resource-limited settings.

The MS formulation of the paper-based test described in this study has two important limitations. First, when visually interpreted, the test cannot differentiate between SCA and other heterozygous forms of SCD (e.g., HbSC). This is because the blood stain pattern produced in paper is determined by the concentration of HbS in the blood sample being tested and is unaffected by the relative proportions of other soluble forms of Hb making up the rest of the sample. As such, the paper-based test would have the highest clinical utility in regions with low SCD genotypic variation (e.g., Angola where the majority of SCD cases are SCA [5]) and lowest utility in regions where other forms of SCD are present (e.g., Burkina Faso and Central West Africa where HbSC is prevalent [34]). Importantly however, we have previously shown that automated image analysis of scanned blood stain patterns can be used to differentiate *HbAS* from *HbSC* (sensitivity of 100%, specificity of 59%) despite human scorers being unable to differentiate the patterns by eye [19]. Second, while the LOD of the MS formulation of the test has been improved to 10% HbS, this LOD may still be insufficient for detecting SCT or SCA in newborns less than six months of age. This is because at birth the cord blood of SCT newborns contains 9.5 ± 4.2% HbA and 6.5 ± 2.8% HbS, and the blood of newborns with SCA contains 10.2 ± 3.9% HbS, meaning that newborns with HbS concentrations near the low end of this physiological range may produce false negative results [35,36]. To address this limitation we have previously developed and validated a separate HS-based test which employs a disposable filter to further improve the LOD of the test, however, this version of the test is currently incapable of differentiating SCT from SCA [20].

In summary, we have improved and characterized the performance of our previously-developed paper-based test for rapid, low-cost, and equipment-free diagnosis of SCA in resource-limited settings. We have demonstrated that the test, compared to the previous version, had a superior limit of detection, faster readout time, better diagnostic performance, longer shelf stability at room temperature, and could be packaged into a self-contained, distributable test kit with a total cost of only $0.21 (USD). A point-of-care test with these qualities represents a significant step towards enabling population-wide screening in resource-limited settings, which, in combination with prompt initiation of prophylactic treatment, could have a transformative impact on the health and well-being of the hundreds of thousands of individuals with SCA born in developing countries each year.

## Figures and Tables

**Table 1:** Detailed cost breakdown of the paper-based SCA screening test kit components.

Test Component	Cost Per Test (USD)
Foil pouch	$0.02
Reagent dropper	$0.05
Blood dropper	$0.08
Chromatography paper	$0.01
Reagent tube	$0.03
Food grade reagents	$0.02
Total	$0.21

## Supplementary Materials

The following are available online at www.mdpi.com/2079-6374/7/3/39/s1: **Figure S1.** Time-lapse of Test from Kit to Readout.
