# Supplementary figures and images for: Substituting Sodium Hydrosulfite with Sodium Metabisulfite Improves Long-Term Stability of a Distributable Paper-Based Test Kit for Point-of-Care Screening for Sickle Cell Anemia
Source: Biosensors (Basel). 2017 Sep 20;7(3):39. doi: 10.3390/bios7030039 (PMC5618045; doi:10.3390/bios7030039)

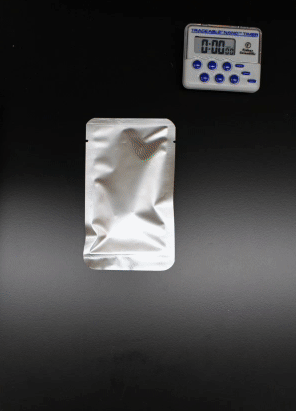

Supplement: Supplementary file 1 [file biosensors-07-00039-s001.zip › biosensors-220072-supplementary.gif]
